# Association of the *IGF1* 5′UTR Polymorphism in Meat-Type Sheep Breeds Considering Growth, Body Size, Slaughter, and Meat Quality Traits in Turkey

**DOI:** 10.3390/vetsci10040270

**Published:** 2023-04-02

**Authors:** Vasfiye Kader Esen, Selim Esen

**Affiliations:** 1 Department of Breeding and Genetics, Sheep Breeding Research Institute, Balıkesir 10200, Turkey; 2 Balikesir Directorate of Provincial Agriculture and Forestry, Republic of Turkey Ministry of Agriculture and Forestry, Balikesir 10470, Turkey

**Keywords:** insulin-like growth factor type-1, growth, carcass, slaughter, sheep, meat-type, body size, SSCP

## Abstract

**Simple Summary:**

Molecular genetic approaches have been developed over the last half-century to identify genetic variation associated with economically important growth performance and carcass characteristics. In the livestock sector, identifying and localizing the genes responsible for each trait, as well as selecting beneficial alleles based on live animal experiments, meat production, and nutritional quality, have played an essential role in improving productivity. The purpose of this study is to examine the association between *IGF1* 5′UTR polymorphisms and various growth and carcass parameters of meat-type sheep breeds raised in Turkey. A total of eight nucleotide changes were identified that were able to characterize three *IGF1* 5′UTR variants, and certain variants were associated with variations in chest width and leg circumference. The *P1* variants had a leaner profile and the *P2* variants had a higher percentage of rack and loin. Nucleotide sequence variations in *IGF1* 5′UTR could, thus, be exploited for marker-assisted selection in order to enhance growth and production attributes and carcass quality.

**Abstract:**

This investigation was conducted to determine how the growth and carcass traits of meat-type sheep breeds raised in Turkey are associated with *IGF1* 5′UTR polymorphisms. Overall, 202 lambs from five breeds were evaluated. We identified eight nucleotide changes (seven substitutions and one deletion) in three variants of *IGF1* 5′UTR by SSCP analysis and nucleotide sequencing. It was found that the *P1* variants had a unique deletion (g.171328230 delT), while the *P2* variants were identified by SNPs rs401028781, rs422604851, and g.171328404C > Y. The *P3* variants possessed one heterozygous substitution (g.171328260G > R) and three homozygous substitutions (g.171328246T > A, g.171328257T > G, g.171328265T > C) not observed in *P1* or *P2*. Based on the growth and production traits, a statistically significant difference was found only in chest width at weaning (*p* < 0.01) and leg circumferences at yearling (*p* < 0.05). The *P1* variants showed a leaner profile with a higher *Musculus longissimus dorsi,* but the differences were not significant (*p* > 0.05). The *P2* variants had a higher percentage of rack (*p* < 0.01) and loin (*p* > 0.05). Moreover, there was no discernible difference between variants, even though the *P3* variants had a higher percentage of neck and leg and the *P1* variants had a higher percentage of the shoulder. It is concluded that nucleotide changes in *IGF1* 5′UTR could be exploited utilizing a marker-assisted selection technique to increase growth and production attributes, as well as carcass quality traits.

## 1. Introduction

A recent report on global population trends stated that the number of people will reach 9.7 billion in 2050 and 10.9 billion in 2100 [1]. As developing countries around the world become more Westernized, the demand for meat, milk, and other animal products is also expected to increase [2]. However, this seems unlikely to be accomplished due to the limited resources available. In addition, it is difficult to control the quality and nutritional value of these protein sources in order to satisfy consumer demands, especially when this goal depends on several factors simultaneously [3]. As an example, meat is affected by a number of factors, including water-holding capacity (WHC), color, tenderness, texture, carcass composition and conformation, animal environments, breeds, stunning, slaughtering, storage conditions, and gene expression [3].

In the livestock sector, advances in molecular genetics have allowed for the identification of genes and sequence variation associated with various production traits [4,5]. Consequently, many scientists are advocating a marker-assisted selection (MAS) program as a means of predicting muscle mass gain in farm animals and determining the most productive individuals [6,7].

One of the promising candidate genes to be used in the MAS program is insulin-like growth factor type-1 (*IGF1*) [8]. A mammal *IGF1* consists of six exons separated by five introns and spans more than 80 kb [9]. While species share a commonality in the sequence and length of exons 1–4, there is considerable variation in exons 5 and 6. The mature *IGF1* peptides that serve as receptor-binding ligands are specified by exons 3 and 4, while exons 1 and 2 determine the type of protein and signal peptides that are encoded for cellular localization following translation [10].

It is widely acknowledged that *IGF1* plays an important role in the regulation of normal growth, placental and fetal development, immunity, reproduction, and metabolism [11,12,13]. In addition, previous studies have shown that the polymorphism in the *IGF1* gene influences growth and production traits in a variety of farm animals, including chickens [14,15], pigs [16,17], goats [18,19], sheep [20,21], and cattle [22,23]. *IGF1* has been shown to stimulate the proliferation and differentiation of myocytes during myogenesis, which may contribute to muscle growth and repair, as well as muscle regeneration and repair [24,25]. Fat deposition and carcass quality in New Zealand Romney sheep have been shown to be affected by six single nucleotide polymorphism (SNPs) in the exon 3, exon 4, and 5′ flanking region of ovine *IGF1* [10]. Similarly, Trukhachev et al. [20] investigated the *IGF1* polymorphism in the Russian Soviet Merino breed and concluded that the identification of SNPs c.-91A > C could be used to predict its parameters of superior meat production. The *IGF1* polymorphism is also associated with lactation persistence in dairy sheep breeds [26], as well as wool and hair production in sheep [27] and goats [28].

A mutation in *IGF1* 5′UTR might possibly have an effect on the functional features of the protein due to the role that it plays in selecting the type of protein and signal peptides that are encoded for cellular localization after translation [29,30]. As discussed in our previous studies [31,32], Kıvırcık (K), Karacabey Merino (KM), German Black-Head Mutton × Kıvırcık (GBK), Hampshire Down × Merino (HM), and Ramlıç (Rambouillet × Daglıç, R) are the main meat-type sheep breeds widely grown in the western part of Turkey in order to meet market demands. To the best of our knowledge, there are no known data in Turkey on the association between the *IGF1* polymorphism with growth, body size, slaughter, and meat quality traits in those meat-type sheep breeds. As a result, the purpose of this study was to determine the impact of the *IGF1* 5′UTR polymorphism on a variety of production variables, including body measurements, slaughter and carcass features, and meat quality.

## 2. Materials and Methods

### 2.1. Ethical Approval

All of the experiments on animals were done in accordance with the Declaration of Helsinki and were approved by the Ethics Committee of the Sheep Breeding Research Institute in Turkey (approval number:13360037). The animals were handled and cared for in the experimental farm unit of the same institute from January to June 2018.

### 2.2. Animals and Feeding Schedules

**Figure 1:**
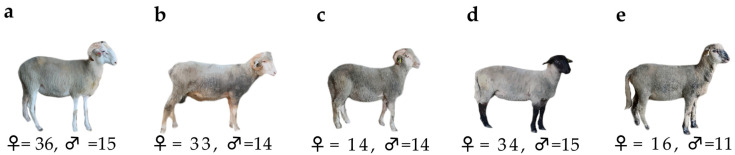
The breeds of sheep used in the study and their distribution by gender: Kıvırcık (**a**), Karacabey Merino (**b**), Ramlıç (**c**), German Black-Head Mutton × Kıvırcık (**d**), Hampshire Down × Merino (**e**).

The animal population and feeding schedules used in the current study were based on our studies that were carried out earlier [31,32,33,34,35]. Figure 1 illustrates the breeds of sheep used in the study and their distribution according to gender. The lambs were kept with their dams in separate barns during the preweaning period, and at 15 days old, they were given access to ad libitum high-quality alfalfa hay and commercial starter feed. Weaned lambs of different breeds were grouped together and fed a diet consisting of concentrated feed (600 g per lamb per day), alfalfa hay (100 g per lamb per day), and a vetches−wheat mixture (300 g per lamb per day) until slaughter. After slaughter, male and female lambs were separated in order to prevent unwanted mating. Between the ages of weaning and yearlings, they were allowed to roam freely on pasture when the weather permitted, and were provided with a diet of alfalfa hay, vetches−wheat mixed hay, concentrate feed, and wheat straw ad libitum. In our previous studies, we provided detailed information about the chemical composition of the roughages and concentrates that were used in this study [31,32].

### 2.3. Measurement of Live Weight, Linear Body Measurements, and In Vivo Ultrasound

Lambs born within 10 days of the end of the lambing season were employed in the study, and they were weaned after a mean of 90.5 ± 5.7 days (mean ± SD) [32]. Within 12 h of birth, the birth weight (BW) was assessed, and on the 90th, 180th, and 360th days of the research, the live weight (LW), linear body measurements, and ultrasound measurements were obtained. LW was recorded before morning feeding to avoid the effects of a full stomach on the accuracy of the measurement. A skilled technician used a flexible calibrated tape and callipers to obtain linear measurements of the lamb while it was standing with its head raised, including the following: body length (BL), back height (BH), rump height (RH), withers height (WH), chest depth (CD), chest width (CW), rump width (RW), chest circumference (CC), leg circumference (LC), and cannon bone perimeter (CBP) [36].

Lambs’ fat thickness (FT), skin thickness (ST), and *Musculus longissimus dorsi* depth (MLDD) were measured between the 12th and 13th ribs using a Mindray DP-20 real-time ultrasound system and Mindray 75L50EAV linear veterinary ultrasound transducers [37]. These measurements were taken after linear body measurements were performed at each specified interval. We used ultrasonic gel as a couplant and manually immobilized the lambs so that we could separate their wool between their 12th and 13th ribs. Taking care not to compress the fat, measurements were collected on the left side, 4 cm from the vertebral column [31]. After the scanned image was taken, MLDD, FT, and ST were measured using the scanner’s electronic callipers.

### 2.4. Assessment of Slaughter and Carcass Characteristics and Meat Quality

Ten male lambs were chosen at random from each breed to be used in our analysis of slaughter and carcass features and meat quality. Before slaughter, the lambs were provided with fresh water and were fasted for 12 h in the institute slaughterhouse. The slaughter weight (SW) of the lambs was noted and then they were slaughtered according to commercial standards. For the purposes of determining the hot carcass weight (HCW), we skinned the animals and then removed the heads, feet, lungs, liver, heart, spleen, gastrointestinal tract, and testicles. The hot dressing percentage (HDP) was calculated by dividing HCW by SW. Following chilling the carcasses at 4 °C for 24 h, the cold carcass weight (CCW) and cold dressing percentage (CDP) were calculated.

Each carcass was split into five portions (neck, shoulder, rack, loin, and leg), and the Longissimus thoracis et lumborum (LTL) muscle was taken between the 5th and 12th thoracic vertebra for additional laboratory investigation [38]. The Fiji image measurement program was used to measure the MLD area (MLDA), MLD perimeter (MLDP), MLD depth (MLDD), MLD width (MLDW), MLD fat thickness (MLD_FT_), the body fatness (BF) of the chilled carcasses (Version 1.52d) [39]. Color parameters of LTL were measured for 0, 48, and 168 h after storage at 4 °C using a portable colorimeter (Chroma Meter CR-410; Konica Minolta, Tokyo, Japan) with three replicates of samples. The filter-paper method was employed to determine WHC, according to Honikel and Hamm [40]. In accordance with previously published studies by Choi and Kim [41] and Gonzales-Barron et al. [42], we evaluated a sample of LTL for thawing loss (TL) and cooking loss (CL), respectively. The shear force (SF) of the cooked LTL samples was measured using a texture analyzer (TA.HDplus, Stable Micro Systems Ltd., Surrey, UK) and Warner Bratzler blade (HDP/WBV, Stable Micro Systems Ltd., Surrey, UK).

### 2.5. Extraction of DNA, Primer Design, PCR Amplification, SSCP Analysis, and Sequencing of DNA

Blood samples were taken from each of the lambs’ vena jugularis and placed into sterile EDTA tubes before the male lambs were slaughtered. Genomic DNA was extracted from blood samples collected from lambs using the GeneAll^®^ DNA extraction kit and Bio-Rad T100 thermal cycler.

Polymorphisms in 265 bp of *IGF1* 5′UTR were discovered to alter growth and carcass features [29,30]; hence, a set of PCR primers designed by Behzadi et al. [29] was employed to amplify this region. Using GeneAll^®^ 2XAmpMaster, DNA was amplified in a 20 µL reaction with each primer weighing 100 ng. The PCR conditions for amplification of the 265 bp promoter region of the *IGF1* 5′UTR were as follows: the initial extension was 3 min at 95 °C, 35 cycles 45 s at 95 °C, 40 s at 60 °C, 50 s at 72 °C, and the final extension was 10 min at 72 °C.

The PCR products were mixed with a loading dye that contained 98% formamide and denatured at 95 °C for 7 min. Following rapid cooling of the samples on wet ice, the samples were separated by acrylamide−bisacrylamide gels (29:11) according to Green and Sambrook [43]. According to the method described by Byun et al. [44], vertical electrophoresis was performed for 4 h at 350 V. As part of the staining technique, the formaldehyde ratio was modified to 2% before determining how visible the electrophoresis bands were.

We utilized a genetic analyzer (ABI PRISM 3500, Applied Biosystems, Foster City, CA, USA) to detect various band patterns within the DNA sequences of the samples. By removing noisy sequences and aligning clear sequences, we were able to visualize the alignment of chromatograms using the Bioedit Sequence Alignment Editor. In order to determine the location of SNPs on the chromosomes, we compared our DNA sequences with those found in the sheep genome (Oar_v3.1, GCA_000298735.1) using the Ensembl Genome Database. Note that two of the SNPs obtained in this study had previously been identified in the reference genome.

According to Falconer and Mackay [45], the genotypes and variant frequencies were calculated for each SNP.

### 2.6. Statistical Analysis

A GLM procedure was employed in Minitab [46] to determine whether the *IGF1* 5′UTR polymorphism influenced the studied traits. To compare LW, body measurements, and in vivo development of MLD throughout different time periods, the fixed effect of breed (K, KM, R, GBK and HM), gender (female and male), type of birth (single and twin), age of dam (2–7+), and variants of *IFG1 promotor-exon 1* (P1-P3; considering one variation at a time), as well as their interactions were taken into account (Model 1). Gender was removed from the model in the analysis of the slaughter and carcass characteristics, retail carcass percentage, and meat quality features, as only male lambs were slaughtered in this study (Model 2). The predicted number of animals for Models 1 and 2 in each group is shown in Table S2 based on the Power analysis of Cohen’s f values (0.10, 0.25, and 0.40 as small, medium, and large values, respectively). To ascertain whether there were statistically significant differences between the color parameters, a repeat-measures analysis of variance was also utilized. Duncan’s multiple range test was used to compare the means, with a significance level of *p* < 0.05 indicating a statistically significant difference.

## 3. Results

In the case of *IGF1* 5′UTR, 21 of the samples did not amplify, which may be the result of a mutation in the major alignment region present in these animals. Thus, 181 animals’ worth of information was used in the association analysis.

**Figure 2:**
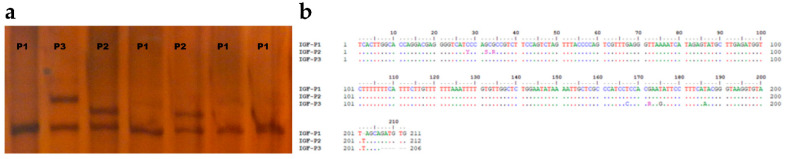
The variation of sequences in meat-type sheep breeds. Different sequences in the region of *IGF1* 5′UTR are found using PCR-SSCP analysis and DNA sequencing (**a**) Alignment of the *IGF1* 5′UTR variant sequences with the NCBI reference sequence *GCA_000298735.1* using the Clustal W algorithm (**b**).

In polyacrylamide gels, three different variants in PCR products were discovered using the PCR-SSCP approach. Three distinct PCR-based SSCP patterns (*P1*, *P2*, and *P3*) were deposited in the NCBI GeneBank OQ197497-OQ197499 (Figure 2a). Alignment of the sanger sequencing’s complementary sequences was performed using the Bioedit’s Clustal W algorithm (Figure 2b).

A comparison of the clear sequences obtained with the reference genome revealed that they were located on the third chromosome and the *IGF1* gene was located within the promoter region of the first exon. On the basis of the difference between the three variants, nucleotide changes were detected in eight nucleotide positions (Table 1). It was only *P1* that showed deletion within the nucleotide sequences (c.202delT). In *P2*, a total of three heterozygous substitutions (g.171328398 G > R(A/G), g.171328400 G > S(G/C), g.171328404 C > Y(C/T)) were observed, of which two had already been identified (rs401028781 and rs422604851). Furthermore, three homozygotes (g.171328246 T > A, g.171328257 T > G, g.171328265 T > C) and one heterozygote (g.171328260 G > R(A/G)) were identified.

The frequency of the wild-type variant (*P1*) was the greatest in each breed and gender (Table 2). The P1 variant was identified in all male and female R lambs. Although neither KM nor HM male lambs carried the *P2* variant, neither did R male lambs. While the *P3* variant was not found in female KM and R lambs, it was also not found in female HM and K lambs. GBK lambs, on the other hand, were the only breed in which all three variants were found in both males and females.

In all breeds, the wild-type variant was more common than the mutant variant in all SNPs, except at position c.202 (Table S2). For all SNP sites in the GBK and KM breeds, two genotypes were observed: wild-type homozygous and mutant heterozygous. Homozygous wild genotypes were found in the R breed at positions c.28, c.32, and c.34, whereas they were found in the HM and K breeds at positions c.167, c.172, c.175, and c.186. The positions of c.8, c.32, and c.34 had the highest frequency of mutant variants in the K breed, whereas positions c.167, c.172, c.175, and c.186 had the highest frequency of mutant variants in the GBK breed. For all breeds except GBK, the deletion rate of the variant T at position c.202 was higher than in the wild type variant. All of the reported SNP variant frequencies in both wild-type and mutant-type homozygotes deviated from the Hardy Weinberg distribution (*p* > 0.05).

**Figure 3:**
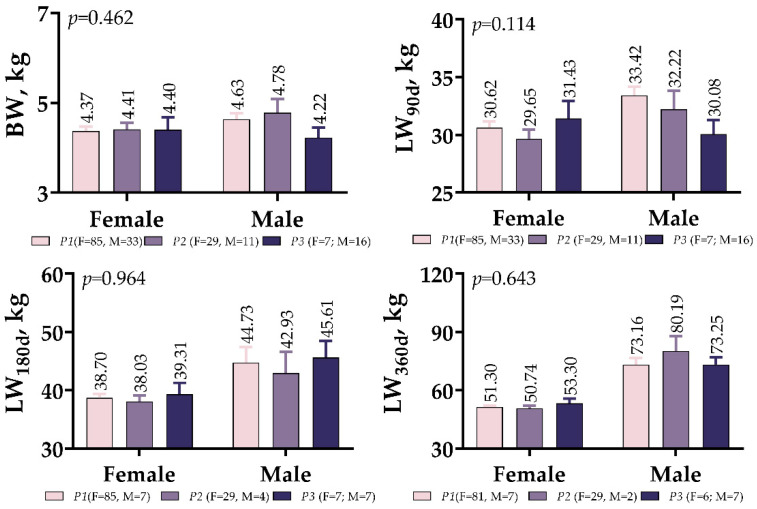
*IGF1* 5′UTR polymorphism in meat-type lambs: effect on weight at different ages.

There were no significant differences between the male and female variants of *IGF1* 5′UTR in terms of BW or LW measured at the different developmental stages (*p* > 0.05, Figure 3). In the *P3* variant, males had a lower BW and weaning weight (LW_90d_) than females, but this changed when measuring 180 days after birth.

Figure S1 offers a visual representation of how the *IGF1* 5′UTR polymorphism influences the body measurements of meat-type lambs. We found statistically significant differences only in CW between the *IGF1* 5′UTR variants at weaning (Figure S1a; *p* < 0.01) and in LC at yearling (Figure S1c; *p* < 0.05). At six months, however, none of the other body measurements had significantly changed (Figure S1b, *p* > 0.05). In the weaning period, males and females of variants *P3* showed the highest and lowest CW values (18.95 cm and 17.14 cm, respectively). It was observed, however, that the difference in CW disappeared after six months. Moreover, the LC of female lambs was comparable between variants; however, the variant *P2* had 24.55–25.57 cm greater LC than the other variants at yearling, and the difference was statistically significant (Figure S1c; *p* < 0.05).

**Figure 4:**
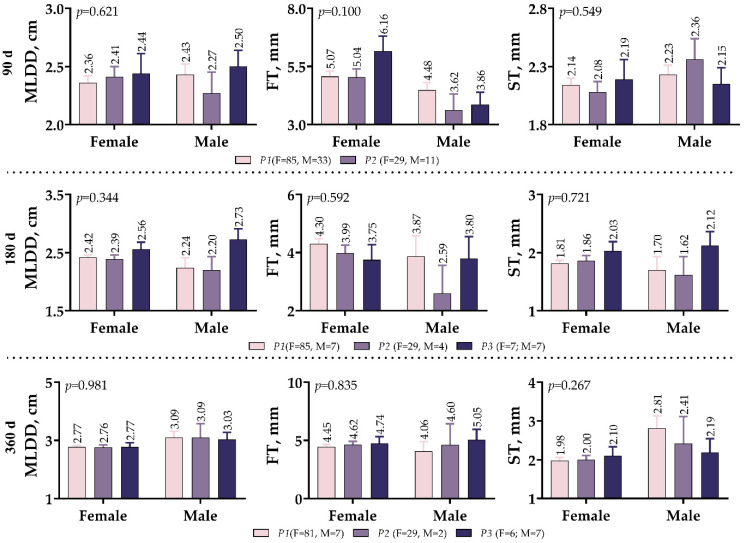
*IGF1* 5′UTR polymorphism in meat-type lambs: effect on Musculus longissimus dorsi muscle development.

As shown in Figure 4, the effects of the *IGF1* 5′UTR polymorphism on the development of MLD in meat-type lambs were not different between males and females. The male variants of *P2* showed the lowest MLDD values at weaning and 180 days (2.27 and 2.20 cm, respectively), while the male variants of *P3* exhibited the highest MLDD values (2.50 and 2.73 cm, respectively). However, in the 360-day measurements, this difference in MLDD between male variants no longer existed. On both the 90th and 180th day measurements, male lambs had lower FT values than female lambs; however, the 360th day measurements were very similar.

**Figure 5:**
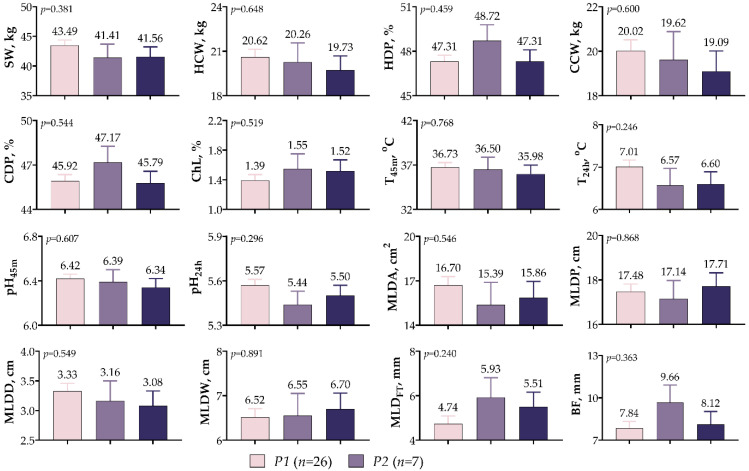
*IGF1* 5′UTR polymorphism in meat-type lambs: effect on carcass traits.

Figure 5 provides an overview of the data from the carcass parameters of the *IGF1* 5′UTR variants. The results indicate that there was no significant relationship between the *IGF1* 5′UTR variants and any of the parameters examined (*p* > 0.05). Although SW and both carcass weights (HCW and CCW) were higher in variant *P1*, the dressing percentages (HDP and CDP) were higher in variant *P2*. In the variants, ChL, T_45m_, T_24h_, pH_45m_, and pH_24h_ values ranged from 1.49–1.55%, 35.98–36.73 °C, 6.57–7.01 °C, 6.34–6.42, and 5.44–5.57, respectively. Likewise, no statistical differences were observed between any of the MLD values obtained using the image processing technique. Compared with the other variants, the *P1* variant had 5.11–7.51% higher MLDD and 5.03–7.84% higher MLDA values and exhibited a leaner profile.

**Figure 6:**
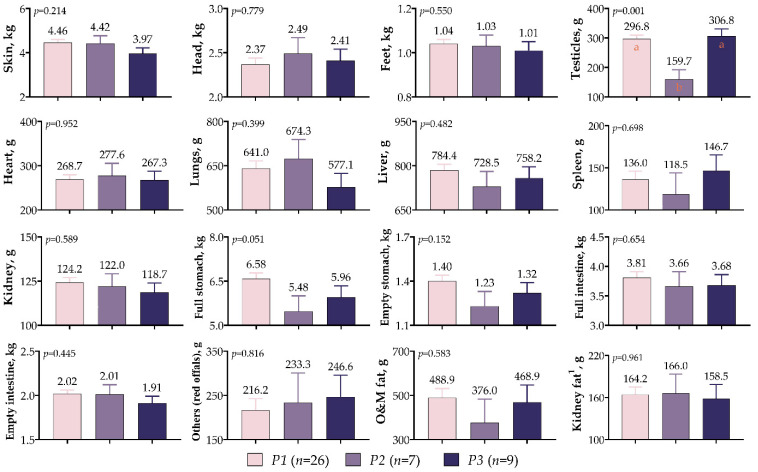
*IGF1* 5′UTR polymorphism in meat-type lambs: effects on non-carcass components. The values with different letters (a–b) in each graph are statistically different (*p* < 0.05).

In Figure 6, it is shown that the effects of the *IGF1* 5′UTR polymorphism on non-carcass components were not significant, except for the weight of the testicles (*p* = 0.001). The mean testicular weight was the lowest for the *P2* variants (159.7 g), while it was similar for the *P1* and *P3* variants (296.8 g vs. 306.8 g). Although not statistically significant, the *P1* variant had the highest weights for skin (4.46 kg), feet (1.04 kg), liver (784.4 g), kidney (124.2 g), full stomach (6.58 kg), empty stomach (1.40 kg), full intestine (3.81 kg), empty intestine (2.02 kg), and omental and mesenteric fat (488.9 g). Furthermore, the *P2* variant was characterized by a head weight of 2.49 kg and a heart weight of 277.6 g, while the *P3* variant was characterized by a spleen weight of 146.7 g and a red offal weight of 246.6 g.

**Figure 7:**
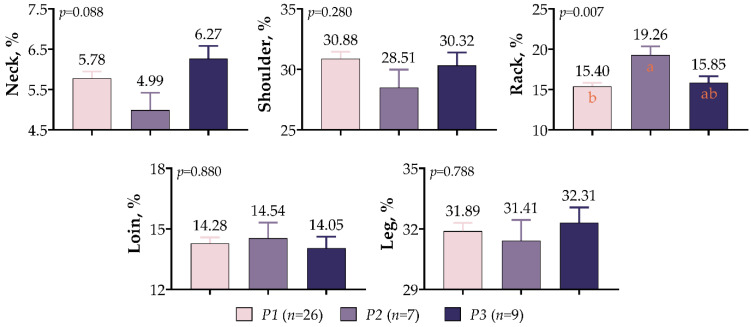
*IGF1* 5′UTR polymorphism in meat-type lambs: effects on retail carcass percentage. The values with different letters (a–b) in each graph are statistically different (*p* < 0.05).

The polymorphisms in *IGF1* 5′UTR had a significant effect only on the rack proportion of meat-type lamb (*p* < 0.01, Figure 7). Lambs of the *P2* variant exhibited higher rack ratios than those of the *P1* and *P3* variants by 25.06% and 21.51%, respectively. There was also a high loin (14.54%) proportion in the *P2* variant; however, the differences were not statistically significant (*p* > 0.05). The difference between variants was insignificant despite the fact that the shoulder proportions were high in *P1* variant lambs, and the neck and leg proportions were high in the *P3* variant lambs (*p* > 0.05).

**Figure 8:**
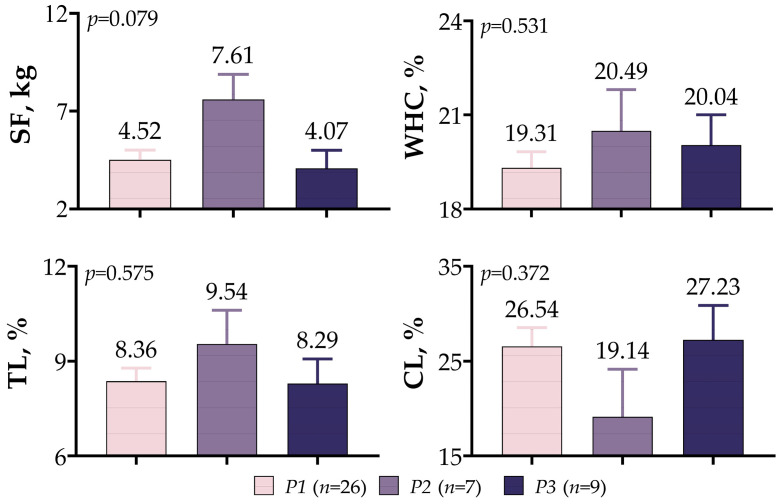
*IGF1* 5′UTR polymorphism in meat-type lambs: effect on meat quality.

A comparison of meat quality assessments in meat-type lambs based on *IGF1* 5′UTR variants is presented in Figure 8. A statistical difference was not observed between variants, despite the fact that SF (7.61 kg), WHC (20.49%), and TL (9.54%) were higher in *P2* variants and CL (27.23%) was higher in *P3* variants (*p* > 0.05).

**Figure 9:**
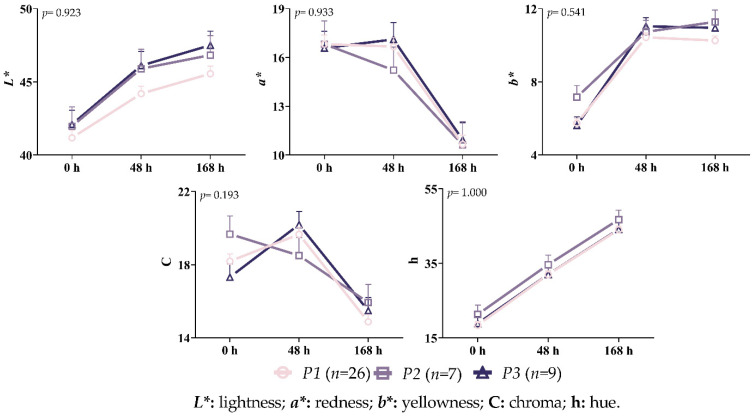
Interaction between variant and time on the color characteristics of the Longissimus thoracis and lumborum muscle during storage.

As can be seen in Figure 9, there were no statistically significant interactions between the genotype and storage time for any of the measured color characteristics (*p* > 0.05). A definite upward trend could be seen in terms of the values of L* and h across all of the variants. Unlike the other variants, a decrease in the a* and C values was observed in the *P2* variant after 48 h of storage. Higher L*, a*, and h values were observed in the *P3* after 168 h of storage, but greater b* and C values were observed in the *P2* variation.

## 4. Discussion

There is compelling evidence that *IGF1*, one of the family of *IGFs*, plays a critical role in cell differentiation, embryogenesis, metabolism, reproductive development, and fetal development, making it a potential candidate gene for characteristics of sheep that are both productive and economical [47]. Yet, there is inconsistent information about the effects of the *IGF1* polymorphism on the growth and production qualities in different sheep breeds. For example, while several studies have associated the *IGF1* polymorphism in the 5′-flanking region with growth traits in Makooei [48], Baluchi [49], and Makui [50] sheep, no such correlations have been discovered in Indian Madras Red sheep [51], Baluchi sheep [52], and Zandi sheep [53]. Moreover, two SNPs in *IGF1 exon 2* (c.144G > A and c.150T > C) and a single SNP in *IGF1 exon 5* (c.495G > A) were demonstrated to be significantly associated with four growth traits (CC at 4 and 9 months of age, BL at 4 months of age, and average daily gain (ADG) from 4 to 9 months of age) in Hulun Buir sheep [21]. In another investigation, Machado et al. [54] found that Santa Inês sheep with *IGF1* haplotype replacements possessed ADG, BL, RH, WH, CC, CW, and LC. In this research, there was a significant difference in CW only during the weaning stage and in LC at the yearling stage between the *IGF1* 5′UTR variants characterized; hence, the *IGF1* polymorphism seen in the *5′*-flanking region and SNPs detected in *IGF1 exon 2* and *IGF1 exon 5* exhibited comparable findings with the studies considered [21,49,50,51]. In female lambs, the g.171328260 G > R(A/G) nucleotide substitution in the *P3* may result in a wider CW, depending on the gender of the individual at weaning. Three heterozygous substitutions in *P2* (g.171328398 G > R(A/G), g.171328400 G > S(G/C), g.171328404 C > Y(C/T)), on the other hand, may have contributed to the increase in LC in adulthood. Oberbauer [55] made the claim that altering the promoter region might have an impact on the structure and operation of the transcription activator binding region of the gene, in line with our findings. However, it should not be assumed that nucleotide modifications in this area have an influence on meat production characteristics, but may be confined as a single component. As part of the same process, other genes and environmental factors must be considered; for instance, McLellan et al. [56] revealed that transcription factors that bind to the E box, such as *MyoD1*, promote the transcription of class 1 transcripts and play a crucial role in muscle cell differentiation.

*IGF1* is an important growth and development regulator in mammalian muscle tissues; together with insulin-like growth factor 2 (*IGF2*), growth hormone (*GH*), and growth hormone-releasing hormone (*GHRH)*, it is an essential component of the somatotropic axis in the development of vertebrates [20]. *IGF1* also plays a role in mediating the stimulatory effects of *GH* and testosterone on muscle growth and development [57]. In the current study, there were no differences in MLDD between weaning (90th day), six months (180th day), and one year (360th day); however, the nucleotide substitutions observed in the *P2* had a significant positive effect on LC. This could be associated with an increase in *IGFI* expression, particularly in the leg muscles. As was previously observed, *IGF1* overexpression was accompanied by an increased expression of the cytoskeletal actin gene, suggesting that *IGF1* stimulates the protein production of structural components [58]. Therefore, the eight nucleotide changes we detected in our study may have caused an increase in LC. Our results were similar to the findings of previous studies showing that *IGFI* mRNA and protein were expressed and up-regulated in C2 and sheep myoblast and satellite cells throughout differentiation [57,59]. Additionally, *IGFI* has been shown to accelerate muscle differentiation after myoblasts exit the cell cycle by activating the *mef2c* and raising myogenin and *MRF4*, resulting in a net increase in structural gene expression and the development of bigger myotubes [60].

In research including Angus, Charolais, and hybrid beef cattle, an SNP (c.512C > T) within the promoter of *IGF1* was investigated to determine the connection between fat deposition and carcass merit [61]. In the Angus cow population, the c.512C > T substitution was found to have a significant impact on carcass average BF, ultrasonography backfat thickness, and carcass lean meat output, with the “CC” genotype had a greater fat depth and lower lean meat yield than the “TT” genotype [61]. Another study revealed that nucleotide sequence in *IGF1 exon 3*, *IGF1 exon 4,* and the flanking region of *IGF1* was associated with the depth of carcass fat at the 12th rib measured by video imaging and the percentage proportion of lean meat in the leg [10]. However, no difference was found in the current study between *IGF1* 5′UTR in MLD_FT_, BF measured after slaughter, and FT values from in vivo ultrasonography measurements taken on days 90, 180, and 360. Thus, the present study differs from those of Islam et al. [61] and Li et al. [10]. One possible explanation is that lambs born at the end of the lambing season were chosen for the research and maintained the same feeding regimen in a considerably more controlled environment. There was also a possibility that the diverse breeds used in this study could have contributed to the outcome, which was in accordance with Li et al. [10].

In a study by Hofmann et al. [62], the effects of maternal dietary restriction during late gestation on the development of female and male reproductive organs were investigated. The results showed that the restricted dam lambs had lower testicles than the lambs born to the control dams. The study also reached the conclusion that maternal dietary restrictions during late gestation decreased *IGF1* mRNA levels during fetal life and interfered with fetal reproductive development, which could have a long-term negative effect on future reproductive success. The results of this study, which examined lambs born to unrestricted dams, showed that lambs with *P2* had considerably reduced testicle weights, highlighting the significance of these genes’ regulated expression for healthy development and reproduction. It is known that the *IGF* system is essential to the proper development of male organs [63]. Therefore, changes in nucleotide substitutions may result in reduced circulating *IGF1* levels and the mRNA expression of insulin-like growth factor 1 receptor (*IGF1R*), resulting in a lower testicular weight. Baker et al. [63] observed comparable results, finding a delay in Leydig cell differentiation causing interstitial space to comprise predominantly undifferentiated mesenchymal cells at 2 weeks after birth in a study evaluating the impact of *IGF1* null mutation on reproductive organs.

The assessment of slaughter and carcass traits on a large scale is both time-consuming and costly. Thus, several researchers have emphasized the importance of molecular markers in order to achieve the breeding objective of improving the carcass quality and weight [31,64]. Previous research has linked carcass weight in sheep [29] and cattle [65] to nucleotide polymorphisms in the *IGF1*, but we did not find such a relationship in our study. This may be explained by differences in the breeds of sheep and the amplified region of the genes used in the study. In contrast, the *IGH1 exon1* polymorphism had a substantial impact on the proportion of rack in a lamb’s carcass. One possible explanation for this might be the up-regulation of the *IGF1* in rack muscle tissue of lambs possessing *P2*. In agreement with our findings, previous research has also shown that the *IGF* family binds to its receptor, modulating the pathways that promote muscle growth and blocking the physiological processes that promote muscle atrophy [66].

Grochowska et al. [64] genotyped the polymorphism (c.654G > A) in exon3 of the *IGF1R* and investigated its association with growth, body size, slaughter, and meat quality traits. They found that the polymorphisms of both *IGF1* and *IGF1R* had a significant impact on the water-holding capabilities of lamb meat. In the current study, we found no significant variations in the evaluation of meat quality or LTL color between the *IGH1 exon1*. These results are consistent, to some extent, with those of Grochowska et al. [64], who found no statistically significant variations between the color parameters of genotypes.

Although there is persuasive evidence that *IGF1* is essential for cell differentiation, embryogenesis, metabolism, and fetal development, our study’s limited sample size for each breed and the fact that 10% of animals did not amplify were major drawbacks. In addition, the fact that the sequence data provide Y, S, and R suggests that SSCP did not discriminate between all of the variants and that *P2* likely represented more than one variation. Many environmental conditions and other genes had a significant influence on determining the features of meat production; hence our study’s findings cannot be regarded as definitive. Therefore, the study findings should be viewed with caution and more research is needed to confirm them, preferably with larger samples and considering additional factors.

## 5. Conclusions

A PCR-SSCP study was undertaken on meat-type sheep breeds raised in Turkey to determine the association between the *IGF1* 5′UTR polymorphism and a number of growth and carcass traits, including body measurements (CW and LC), MLD development, non-carcass components (testicles weight), and retail carcass percentage (rack percentage). The results show that nucleotide changes on *IGF1* 5′UTR may be used in a MAS strategy to improve the sheep carcass quality characteristics, and that differences between variants can be recognized. Furthermore, it is recommended that studies in larger populations be conducted to confirm the results obtained.

## Figures and Tables

**Table 1:** Variation in the sequence of *IGF1* 5′UTR.

#	Position ^1^	Nucleotide Sequences			Chromosome Location ^2^	SNP rs ID
		*P1*	*P2*	*P3*		
1	c.28	C	Y(C/T)	C	3:171,328,404	-
2	c.32	G	S(G/C)	G	3:171,328,400	rs.401028781
3	c.34	G	R(A/G)	G	3:171,328,398	rs.422604851
4	c.167	T	T	C	3:171,328,265	-
5	c.172	G	G	R(A/G)	3:171,328,260	-
6	c.175	T	T	G	3:171,328,257	-
7	c.186	T	T	A	3:171,328,246	-
8	c.202	-	T	T	3:171,328,230	-

^1^ Positions are numbered according to HGVS. ^2^ Chromosome locations are given according to the Oar_v3.1 (GCA_000298735.1).

**Table 2:** Frequency of *IGF1* 5′UTR variants by race and gender.

Breed	*n*	Variant		
		*P1*	*P2*	*P3*
Kıvırcık	44	0.64	0.36	-
*Female*	31	0.68	0.32	-
*Male*	13	0.54	0.46	-
Karacabey Merino	44	0.75	0.09	0.16
*Female*	31	0.87	0.13	-
*Male*	13	0.46	-	0.54
Ramlıç	24	0.79	-	0.21
*Female*	13	1.00	-	-
*Male*	11	0.55	-	0.45
German Black-Head Mutton × Kıvırcık	46	0.48	0.28	0.24
*Female*	31	0.51	0.26	0.23
*Male*	15	0.40	0.33	0.27
Hampshire Down × Merino	23	0.70	0.30	-
*Female*	15	0.53	0.47	-
*Male*	8	1.00	-	-

## Data Availability

Not applicable.

## Supplementary Materials

The following supporting information can be downloaded at: https://www.mdpi.com/article/10.3390/vetsci10040270/s1. Table S1: Sample size for multigroup comparisons for optimum animal research. Table S2: Genotype and variant frequencies of *IGF1* 5′UTR. Figure S1: The effect of *IGF1* 5′UTR polymorphism on body measurements in meat-type lamb at various times [67].
