# Supplementary material for: Association of the IGF1 5′UTR Polymorphism in Meat-Type Sheep Breeds Considering Growth, Body Size, Slaughter, and Meat Quality Traits in Turkey
Source: Vet Sci. 2023 Apr 2;10(4):270. doi: 10.3390/vetsci10040270 (PMC10146731; doi:10.3390/vetsci10040270)
Supplement: Supplementary file 1 [file vetsci-10-00270-s001.zip › vetsci-2257997-supplementary.pdf]

Association of the *IGF1* 5′UTR Polymorphism in Meat-Type Sheep Breeds considering Growth, Body Size, Slaughter, and Meat Quality Traits in Turkey

Vasfiye Kader Esen [1,*] and Selim Esen [2,*]

[1] Department of Breeding and Genetics, Sheep Breeding Research Institute, Balıkesir 10200, Turkey
[2] Balikesir Directorate of Provincial Agriculture and Forestry, Republic of Turkey Ministry of Agriculture and Forestry, Balikesir 10470, Turkey
* Correspondence: vasfiye.esen@gmail.com (V.K.E.); selim_esen01@hotmail.com (S.E.)

Table S1. Sample size for multigroup comparisons for optimum animal research[1].

Model	Power	Breed	Gender	Type of Birth	Age of Dam	Variants	Total Animals			Animals per Group		
							Small	Medium	Large	Small	Medium	Large
1	0.80	+	+	+	+	+	7560	1440	NA	21	4	NA
	0.90	+	+	+	+	+	9000	1800	1080	25	5	3
2	0.80	+		+	+	+	5400	1080	540	30	6	3
	0.90	+		+	+	+	6480	1260	540	36	7	3

[1] Cohen's f vulues of 0.10,0.25, and 0.40 were selected to represent small, medium, and high effect sizes, respectively. Breed: Kıvırcık, Karacabey Merino, Ramlıç, German Black-Head Mutton × Kıvırcık, Hampshire Down × Merino; Gender: male, female; Type of Birth: single, twin; Age of Dam: 2, 3, 4, 5, 6, 7+; Variants: *P1*, *P2*, *P3*; NA: not adjusted; +: participated in the model. Statistical power analyses were done using the "pwr" package in the R program, as suggested by Cohen [1].

Table S2. Genotype and variant frequencies of *IGF1* 5'UTR.

#	Position[1]	Breed	n	Genotype Frequency		Variant Frequency	
				**CC**	**CT**	**C**	**T**
1	c.28	K	44	0.64	0.36	0.82	0.18
		KM	44	0.91	0.09	0.95	0.05
		GBK	46	0.72	0.28	0.86	0.14
		HM	23	0.70	0.30	0.85	0.15
		R	24	1.00	0.00	1.00	0.00
				**GG**	**GC**	**G**	**C**
2	c.32	K	44	0.64	0.36	0.82	0.18
		KM	44	0.91	0.09	0.95	0.05
		GBK	46	0.72	0.28	0.86	0.14
		HM	23	0.70	0.30	0.85	0.15
		R	24	1.00	0.00	1.00	0.00
				**GG**	**AG**	**G**	**A**
3	c.34	K	44	0.64	0.36	0.82	0.18
		KM	44	0.91	0.09	0.95	0.05
		GBK	46	0.72	0.28	0.86	0.14
		HM	23	0.70	0.30	0.85	0.15
		R	24	1.00	0.00	1.00	0.00
				**TT**	**TC**	**T**	**C**
4	c.167	K	44	1.00	0.00	1.00	0.00
		KM	44	0.84	0.16	0.92	0.08
		GBK	46	0.76	0.24	0.88	0.12
		HM	23	1.00	0.00	1.00	0.00
		R	24	0.79	0.21	0.90	0.10
				**GG**	**AG**	**G**	**A**
5	c.172	K	44	1.00	0.00	1.00	0.00
		KM	44	0.84	0.16	0.92	0.08
		GBK	46	0.76	0.24	0.88	0.12
		HM	23	1.00	0.00	1.00	0.00
		R	24	0.79	0.21	0.90	0.10
				**GG**	**GC**	**G**	**C**
6	c.175	K	44	1.00	0.00	1.00	0.00
		KM	44	0.84	0.16	0.92	0.08
		GBK	46	0.76	0.24	0.88	0.12
		HM	23	1.00	0.00	1.00	0.00
		R	24	0.79	0.21	0.90	0.10
				**GG**	**GC**	**G**	**C**
7	c.186	K	44	1.00	0.00	1.00	0.00
		KM	44	0.84	0.16	0.92	0.08
		GBK	46	0.76	0.24	0.88	0.12
		HM	23	1.00	0.00	1.00	0.00
		R	24	0.79	0.21	0.90	0.10
				**TT**	**-**	**T**	-
8	c.202	K	44	0.36	-	0.36	-
		KM	44	0.25	-	0.25	-
		GBK	46	0.52	-	0.52	-
		HM	23	0.30	-	0.30	-
		R	24	0.21	-	0.21	-

K: Kıvırcık, KM: Karacabey Merino, R: Ramlıç, GBK: German Black-Head Mutton × Kıvırcık, HM: Hampshire Down × Merino.

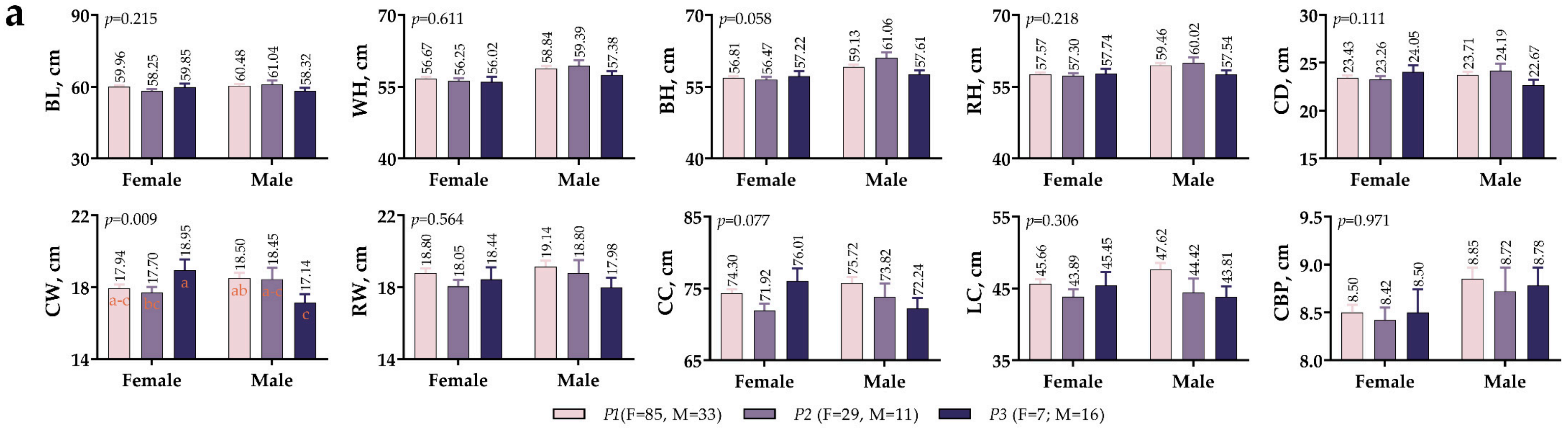

Figure S1. *IGF1* 5'UTR polymorphism in meat-type lambs: effect on body measurements. a: Body measurements on the 90th day. The values with different letters (a-c) in each graph are statistically different ($p < 0.05$).

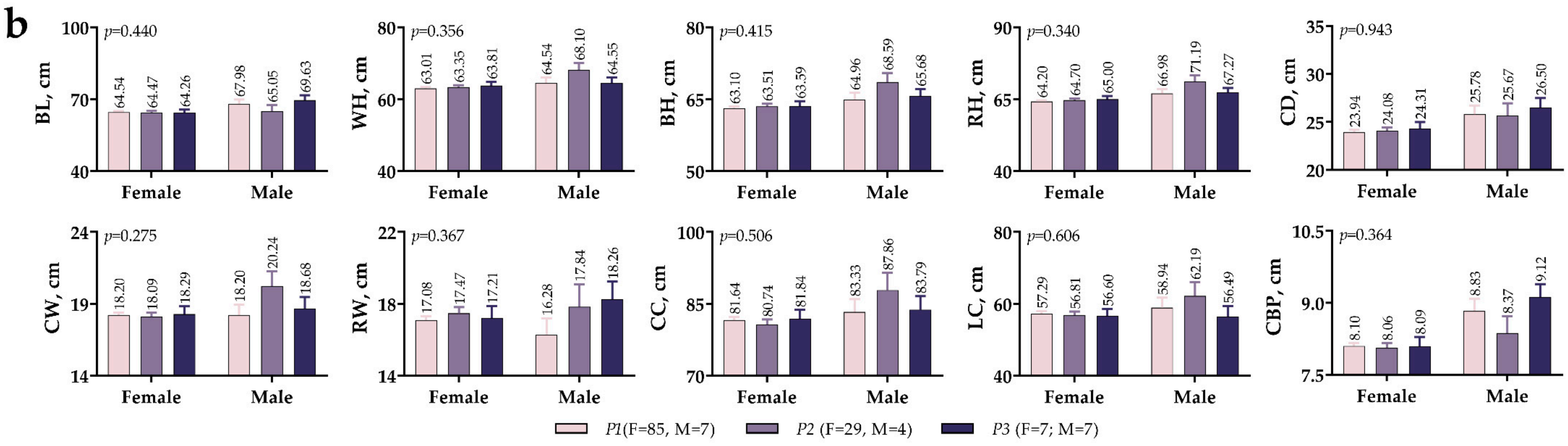

Figure S1 continued. *IGF1* 5'UTR polymorphism in meat-type lambs: effect on body measurements. b: Body measurements on the 180th day.

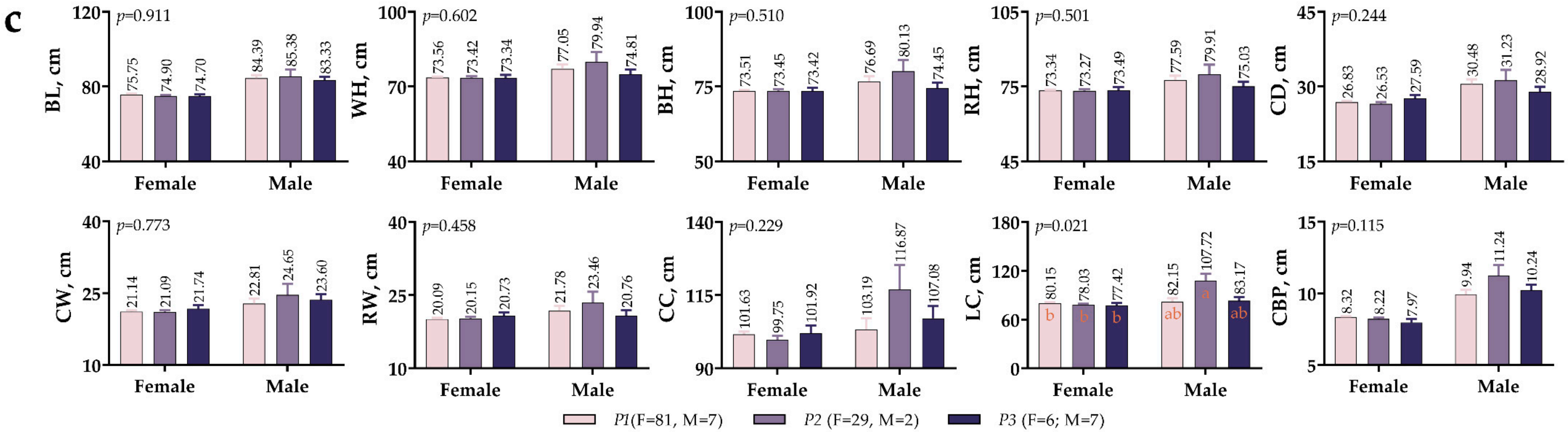

Figure S1 continued. *IGF1* 5'UTR polymorphism in meat-type lambs: effect on body measurements. c: Body measurements on the 360th day. The values with different letters (a-c) in each graph are statistically different ($p < 0.05$).

References

1. Cohen, J. Statistical power analysis for the behavioral sciences (2nd ed.); Hillsdale,NJ: Lawrence Erlbaum, 1988.
